# Down-Regulation of *CYP79A1* Gene Through Antisense Approach Reduced the Cyanogenic Glycoside Dhurrin in [*Sorghum bicolor* (L.) Moench] to Improve Fodder Quality

**DOI:** 10.3389/fnut.2019.00122

**Published:** 2019-08-30

**Authors:** Arun K. Pandey, Pusuluri Madhu, Basrur Venkatesh Bhat

**Affiliations:** ^1^ICAR-Indian Institute of Millets Research (IIMR), Hyderabad, India; ^2^International Crops Research Institute for the Semi-Arid Tropics (ICRISAT), Hyderabad, India

**Keywords:** dhurrin, HCN, CYP79A1, antisense, *Sorghum bicolor*, transgenics, forage quality

## Abstract

A major limitation for the utilization of sorghum forage is the production of the cyanogenic glycoside dhurrin in its leaves and stem that may cause the death of cattle feeding on it at the pre-flowering stage. Therefore, we attempted to develop transgenic sorghum plants with reduced levels of hydrogen cyanide (HCN) by antisense mediated down-regulation of the expression of cytochrome P450 CYP79A1, the key enzyme of the dhurrin biosynthesis pathway. *CYP79A1* cDNA was isolated and cloned in antisense orientation, driven by rice *Act1* promoter. Shoot meristem explants of sorghum cultivar CSV 15 were transformed by the particle bombardment method and 27 transgenics showing the integration of transgene were developed. The biochemical assay for HCN in the transgenic sorghum plants confirmed significantly reduced HCN levels in transgenic plants and their progenies. The HCN content in the transgenics varied from 5.1 to 149.8 μg/g compared to 192.08 μg/g in the non-transformed control on dry weight basis. Progenies with reduced HCN content were advanced after each generation till T_3_. In T_3_ generation, progenies of two promising events were tested which produced highly reduced levels of HCN (mean of 62.9 and 76.2 μg/g, against the control mean of 221.4 μg/g). The reduction in the HCN levels of transgenics confirmed the usefulness of this approach for reducing HCN levels in forage sorghum plants. The study effectively demonstrated that the antisense *CYP79A1* gene deployment was effective in producing sorghum plants with lower HCN content which are safer for cattle to feed on.

## Introduction

The sorghum crop [*Sorghum bicolor* (L.) Moench] is grown worldwide in 45.38 million hectares and accounts for the production of 6.37 million tons of grains (1). Nearly 80% of the cultivated area lies in Asia and Africa. It is the fifth major cereal crop in the world, mainly cultivated in semi-arid regions. Sorghum is an important grain and forage crop of semi-arid regions due to its high adaptability and suitability to rain-fed low input agriculture. One of the major factors limiting the utilization of sorghum forage, especially till the flowering stage, is the production cyanogenic glycoside, i.e., dhurrin, which is toxic to the feeding livestock (2). Hydrocyanic acid (HCN) toxicity due to hydrolysis of dhurrin from sorghum forage in the rumen of the cattle causes their death. Leaves and stems of all sorghum species contain dhurrin. Other plants also produce HCN, but in lesser amounts, whereas in sorghum it is produced in larger amount which is hazardous to the animal species (3).

Sorghum seedling synthesizes the cyanogenic glycoside dhurrin (β-D-glucopyrinosyloxy-(S)-p-hydroxy-mandelontrile) using L-tyrosine as the precursor amino acid (4). Tyrosine is converted to p-hydroxyphenyl acetaldoxime by two multifunctional cytochrome P450s (CYP79A1, CYP71E1) each encoded by a single gene (5). CYP79A1 catalyes two consecutive N-hydroxylation reactions, followed by a dehydration and decarboxylation reaction (6) where conversion of tyrosine to p-hydroxyphenyl-acetaldoxime occurs (5, 6). The oxime is then converted by CYP71E1 to the aglycon p-hydroxymandelonitrile (5, 7) which is subsequently acted upon by a soluble UDPG-glycosyl transferase to produce dhurrin (5, 8). Sorghum mutants were identified through chemical mutagenesis which are deficient in the production of dhurrin and cyanide release (9). One of these mutations t*cd1* had a lowered activity of CYP79A1, the first enzyme in dhurrin production. This behaved like a recessive allele and followed a Mendelian pattern of inheritance. Though cyanogenesis often appears to follows classical Mendelian inheritance, its quantitative expression at a particular point in space and time is highly plastic (10). The Caudatum group of sorghums exhibited the highest and the Guinea group showed the least dhurrin content in the sorghum leaf (11). They suggested that genes for both biosynthesis and catabolism are important in determining natural variation for leaf dhurrin in sorghum in different environments.

This investigation was aimed at obtaining sorghum plants with reduced expression levels of the gene *CYP79A1*, which is responsible for the production of cyanogenic glycosides, using the antisense approach. This approach was thought to be feasible and effective, and has been shown to reduce linamarin in cassava (12) and obtain cassava plants with 94% reduction in cyanogen production in transgenics with anti-sense of *CYP79D1* and *CYP79D2* genes that regulate the cyanogenesis pathway in cassava. Moreover, the effectiveness of these sorghum genes inducing cyanogen production has been demonstrated in arabidopsis and tobacco (13). Other robust tools such as RNA interference-mediated down-regulation technologies (14) were not deployed since it was not intended to completely block the dhurrin biosynthesis pathway as a small quantity of the dhurrin may be desirable as a defense against insects (15). Antisense technology is known to substantially down-regulate but not block the target gene completely (12, 16, 17). For sorghum, the *CYP79A1* gene is the candidate of choice as it is the first enzyme involved in the dhurrin biosynthesis pathway and the down-regulation of *CYP79A1* would lead to no accumulation of secondary products.

## Materials and Methods

### Gene Isolation and Development of the Antisense–Vector

Based on the sequence of the *CYP79A1* cDNA sequence [Genbank ID U32624; Koch et al. (5)], primers were designed (forward primer CYLS1 5′- ATG GCG ACA ATG GAG GTA GAG GCC GCG GCC G-3′ and reverse primer CYLS2 5′- TCA GAT GGA GAT GGA CGG GTA GAG GTG CG-3′) to amplify the largest ORF (1.67 kb) of the *CYP79A1* gene. From the RNA of the leaf tissue of sorghum genotype CSV 15, *CYP79A1* cDNA was synthesized and amplified by RT-PCR (OneStep RT-PCR kit, Qiagen). The amplicon was cloned in the pTZ57R/T vector (Fermentas, USA) and confirmed by sequencing. The clone with antisense orientation with regards to T7 promoter (*Sma*I-*Xba*I in antisense direction) was selected for sub-cloning in pJS108.

**Figure 1:**
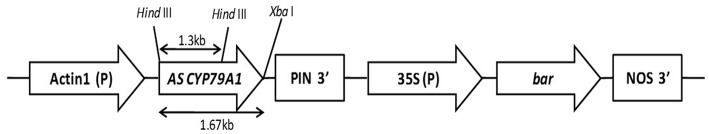
Partial map of *CYP79A1* Antisense RNA construct (pJS108-*CYP79A1*-*AS*) Vector backbone from plasmid pJS108 (courtesy: Dr. Ray Wu, Cornell University, USA); Act1- Promoter of rice actin (*Act 1*) gene; A.S *CYP79A1*- cDNA of *CYP79A1* in antisense orientation; 35S—CaMV35S promoter; *bar* encodes the enzyme Phosphinothricin acetyl transferase that confers Basta resistance; PIN and NOS—termination sequences.

The ORF of *CYP79A1* was excised out from the pTZ57R/T vector at the restriction enzyme sites of *Xba*I and *Sma*I and subcloned into the pJS108 vector. The *UidA* gene was excised out of pJS108 using *Hind*III and *Xba*I. The *Hind*III restricted cohesive terminus (at 5′ end) was filled using T_4_ DNA polymerase to create a blunt end. Thus, the vector had an *Xba*I terminus and a blunt end and the *CYP79A1* fragment (to be inserted) had a blunt end (3′ end) and an *Xba*I terminus (5′ end). Semi-directional cloning was hence possible leading to the antisense orientation of the insert. The ligated vector was transformed into *E. coli* and designated pJS108-*CYP79A1*-AS. Restriction analysis of the plasmid from the *E. coli* confirmed the presence and the antisense orientation of the *CYP79A1* fragment in the plasmid (Figure 1).

### Genetic Transformation in Sorghum to Generate the Putative Transgenic

Shoot apices were obtained from aseptically germinated seedlings of dual-purpose (grain and fodder purpose) sorghum variety *CSV 15*. A cut was made at the base of the seedling apex, just below the attachment of the largest expanded leaf. The top portion with the parts of the unexpanded primordial leaves intact was used as explant (18). The pH of the Murashige and Skoog basal medium was adjusted to pH 5.8. Before bombardment, explants were placed on osmotic medium (MS salts + 17.7 μM BAP + 2.3 μM kinetin + 0.9 μM 2, 4-D + 0.4 M mannitol +0.4 M sorbitol) for 4 h. Twenty-five to thirty explants were placed on each culture plate (94 mm diameter) for bombardment. A home-made particle inflow gun (PIG) was used for gene transfer (19). Genetic transformation of the explants was carried out using tungsten particles (1.0–1.5 μm) coated with the plasmid (1 μg/μl) at a ratio of 1:1 (5 μl each) under a helium gas pressure of 12 kg/cm^2^ and partial vacuum (600 mm Hg). The bombarded explants were then left on the osmotic medium and incubated in culture room at 25°C for 24 h in 16 h light/8 h dark condition.

The bombarded explants were sub-cultured on the somatic embryo induction medium (MS salts + 17.7 μM BAP + 2.3 μM kinetin + 2.2 μM 2, 4-D). After 2 weeks in the medium, the enlarged primordial leaves were trimmed while retaining the bulged shoot apex. These explants were further sub-cultured for 2 weeks on somatic embryo maturation medium (MS salts + 17.7 μM BAP + 2.2 μM 2, 4-D). After 2 weeks, the meristematic masses containing multiple buds were dissected into 2 to 3 pieces and sub-cultured on somatic embryo germination medium (MS basal + 17.7 μM BAP + 1.3 μM 2,4-D + 7.57 μM Basta), where the multiple bud initials develop into plantlets in 2 weeks. A two-step selection strategy was imposed using Basta @ 7.57 μM for the first 20 days followed by Basta @ 15.14 μM with sub-culturing after every 10 days in each medium, thus imposing selection for 40 days in total. Surviving shoot buds were transferred to shoot elongation medium (MS basal + 4.4 μM BAP + 2.45 μM IBA) and sub-cultured every 2 weeks. All growth stages since bombardment were maintained at 25°C in 16 h light/8 h dark conditions until hardening and transfer to the glasshouse. The healthy shoots were transferred to hormone-free MS medium until they set roots. Later they were transferred to pots in glasshouse and raised to maturity.

### Molecular Characterization of Generated Transgenic Plants

#### Polymerase Chain Reaction (PCR)

PCR analysis was done to verify the presence of the *bar* gene in the regenerated plants. Genomic DNA was isolated from the leaves of one-month old transformants by CTAB method (20). The presence of *bar* transgene was determined by PCR using 250 ng of genomic DNA, deploying 1.0 μM of each of forward primer PAT-F (5′-ACC ATC GTC AAC CAC TAC ATC G-3′) and reverse primer PAT-R (5′-TCT TGA AGC CCT GTG CCT C-3′), in 20 μl reaction containing 1X PCR buffer (Fermentas), 1.2 μM MgCl_2_, 0.5 μM dNTPs (Fermentas) and 2.5 units of Taq DNA polymerase (Fermentas). The first 10 cycles consisted of denaturation for 1 min at 95°C, followed by annealing for 1 min at 60°C in the first cycle, reducing by 1°C each cycle (Touchdown PCR), and extension for 1.5 min at 72°C. This was followed by 25 cycles of 1 min at 95°C, 1 min at 50°C, and 1.5 min at 72°C. Final extension was done at 72°C for 10 min.

#### Southern Hybridization

Southern blot hybridization was carried out using 20 μg of genomic DNA from leaves of 1 month old transformed plants according to the standard protocol (21). To check for transgene integration, the *Hind*III enzyme that releases the major fragment (1.3 kb) of *CYP79A1* antisense gene was deployed. To estimate the number of sites of integration of transgenes, the enzyme *Xba*I that has a single restriction site in the plasmid was used. The digested DNA was separated by electrophoresis and transferred to a nylon membrane (Hybond-N^+^, Amersham) by vacuum blotting. The probe for verifying transgene integration was prepared by digesting pJS108-*CYP79A1*-AS with *Hind*III. The 1.3 Kb fragment was purified with Qiagen Gel extraction kit and used as probe. Probe for copy number was prepared through PCR. The *bar* gene probe was amplified by PCR using plasmid (pJS108-*CYP79A1*-AS), deploying forward primer PAT-F and reverse primer PAT-R as done in PCR analysis. The length of the probe was 294 bp. The membrane was probed with respective P^32^dCTP radioactive labeled probes as per manufacturer's instructions (NEBlot Kit, New England Biolabs). The hybridization of the probe was detected on X-ray film by autoradiography.

#### Reverse Transcription Polymerase Chain Reaction (RT-PCR)

The mRNA was isolated from putative transgenic using the PolyATract® mRNA isolation system III (Promega). RT-PCR amplification was performed in a total volume of 20 μl containing 1X OneStep RT-PCR buffer (Qiagen), dNTPs 10 mM, 5X Q Solution (Qiagen), 1.0 μM of each primer (PAT-F and PAT-R), 1.0-unit Reverse Transcriptase enzyme mix (Qiagen) and 300 ng of mRNA. For reverse transcription reaction, the mix was incubated at 55°C at 30 min followed by initial PCR activation at 95°C for 15 min. The touch-down PCR was performed as described in the PCR section above. RT-PCR was also done to amplify the *CYP79A1* antisense cDNA product whose sequence was found to be identical to the corresponding reverse sequence of a native *CYP79A1* fragment.

#### Quantitative Real Time PCR (qRT-PCR)

Total RNA was isolated from leaf tissues of T_4_ generation plants originating from two insertion events using RNeasy Plant Mini kit (Qiagen) and treated with DNase according to the manufacturer's protocol. 4 μg of RNA was reverse transcribed using Superscript III (Invitrogen) and RNase H treated according to the protocol. A 1:3 dilution of the cDNA was used for quantitative PCR performed on a LightCycler 480 II machine (Roche) using LightCycler 480 SYBR Green I Master Kit (Roche) according to the manufacturer's protocol. For assaying CYP79A1, primers 5′- ATGGCGACAATGGAGGTAGA-3′ and 5′-CACCTCCGGGTTGTTCACCA-3′ were used. Reference gene 18s rRNA (22) was used as internal control with primers 5′- GGCTCGAAGACGATCAGATACC-3′ and 5′- TCGGCATCGTTTATGGTT-3′. The relative expression analysis module of LightCycler 480 software release 1.5 was used adopting the cDNA from non-transformed control as reference. Melt curve analyses were done to ensure the absence of multiple peaks and primer dimers. The experiment was performed in three technical replicates.

### Phenotyping of Generated Transgenic to Estimate the Level of Hydrogen Cyanide (HCN)

Samples were collected and HCN content was analyzed 45 days after sowing (23). Whole plants excluding root portion were collected (three biological replicates) and were finely chopped using a scalpel blade. 1.0 g of finely chopped plant material was poured immediately into 15 ml capacity air-tight glass test tube and 200 μl of chloroform was added. A strip of Whatman No. 1 filter paper (1 cm × 10 cm size) that was pre-dipped in saturated alkaline picric acid was stuck to the cork stopper and immediately suspended in the air-tight tube by closing the cork stopper. This test tube with chopped plant sample and filter paper strip was kept at room temperature for 24 h. Next day the filter paper strip was placed in another tube with 10 ml of distilled water and was washed in water carefully by vortex until all color of the strip comes into solution. Optical density of the solution was measured at 515 nm using a spectrophotometer (Multiscan spectrum, Thermo). Another set of filter paper strips were introduced in the air-tight tube with plant samples and assayed as before after 48 h. Standard curve was prepared with the help of KCN. HCN content was calculated using the standard curve. The percent dry matter in a fresh sample was separately estimated after drying in oven, to express HCN content on dry weight basis.

### Visual Phenotyping of Generated Transgenic Plants Through Leaf Painting Assay

Transgenic plants were characterized in terms of sensitivity to the DL-Phosphinothricin (PPT) swab tests performed 25 days after sowing. For leaf painting, a line was drawn on the upper, fully emerged leaves using a black permanent marker. PPT solution (0.5 mg/ml) containing 0.01% Tween-20 (wetting agent) was painted below the line and toward the leaf tip with the help of cotton swab. The leaves were scored for symptoms of leaf scorching after 48 h of painting (24). Symptoms of PPT glutamine synthetase damage manifest as chlorotic discoloration giving a scorched appearance (Figure S1). Those plants with <50% leaf area scorched were classified as resistant (due to *bar* transgene expression). All the control plants recorded scorching in 90–100% leaf area. The non-transformed plants of tissue culture origin were used as control.

## Results

Reduced levels of HCN is required to improve fodder quality. Through genetic modification we are able to achieve this goal.

### Particle Inflow Gum Mediated Genetic Transformation and Generation of Putative Transgenics With Reduced HCN

**Figure 2:**
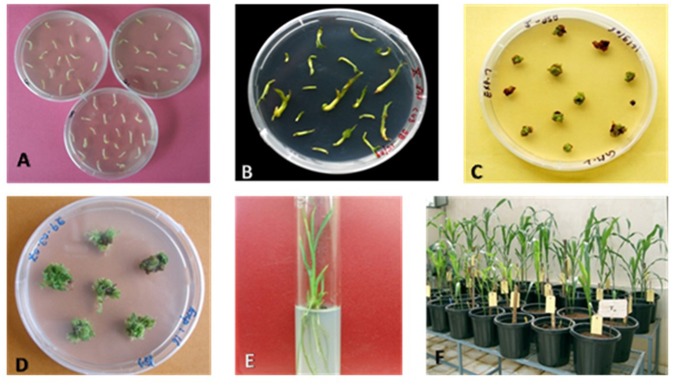
Different steps of Sorghum transformation to generate putative transgenices. **(A)** Shoot meristem explants on induction medium; **(B)** Growth and bulging of explants on induction medium after 12–14 days of incubation; **(C)** Survival of transformed explants in selection; **(D)** Explants with shoot formation in elongation medium; **(E)** Well-developed plants in the rooting medium; **(F)** Putative transgenic plants acclimatized in the glasshouse.

Particle bombardment is an efficient method of genetic transformation of cereals, wherein biological molecules are driven at high velocity into the target tissue. It offers advantages like the introduction of multiple genes, the simplicity of introducing genes, and transformation in those plants, where agro-infection is difficult. Genetic transformation of sorghum using shoot meristem explants was carried out, using tungsten particles (1–1.5 μm diameter) coated with the *CYP79A1* antisense RNA construct (pJS108-*CYP79A1-AS*) (Figure 1) under a helium gas pressure of 12 kg/cm^2^ and partial vacuum (600 mm Hg). The bombarded explants were then left on the induction medium followed by selection (Figure 2A).

*In vitro* selection against the selectable marker (*bar*) using Basta was initiated 4 weeks after bombardment. The 4 weeks incubation period allowed for the recovery and proliferation of the cells transformed by bombardment, which might have been damaged during the process of delivering DNA. The meristems enlarged while keeping in the induction and maturation media (Figure 2B). After 2 weeks, the meristematic masses containing multiple buds were allowed to develop into auxiliary plantlets on somatic embryo germination medium with selection agent Basta for 40 days. Only one-sixth of the transformed explants (2,400 in total) survived Basta selection (Figures 2C,D). Transformed shoot forming sectors could be seen as green to yellow colored growing tissue against a background of brown to black-colored necrotic tissue and the regenerated shoots. The surviving shoots were transferred to hormone-free MS medium to set roots (Figures 2E,F).

### Confirmation of Transgenics Using Different Molecular Biology Techniques

Stable integration and effective expression of foreign genes are of critical importance for the successful application of genetic engineering in agricultural crops. The most common and powerful ways of detecting the foreign DNA in a transgenic are PCR and Southern analysis. The expression of transgene may be verified by RT-PCR and assaying for expected biochemical changes in traits such as cyanogenic potential.

#### Polymerase Chain Reaction (PCR) and Southern Blotting-Based Confirmation of Putative Transgenics

**Figure 3:**
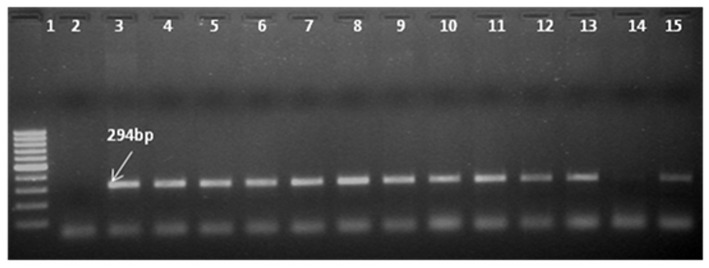
Identification of transgene presence in T_0_ plants by Polymerase Chain Reaction (PCR) with primers specific to *bar* gene shows amplification of 294 bp product. Ingel **Lane 1** signifies 100 bp DNA ladder (Fermentas). **Lane 2** and **3** designate negative control and positive control. Plasmid DNA of construct employed in transformation was used as positive control and genomic DNA of untransformed plant was used as negative control. **Lanes 4–15** indicate the line number of respective T_0_ plants (S18, S28, S13, S6, S14, S32, S17, S31, S27, S25, S29, S9, S11).

PCR was used to confirm the presence of the transgene in the regenerated plants and their progenies till T_3_ generation. Since both the *CYP79A1* native sense and antisense sequences are likely to give the same amplification, PCR analysis was done to verify the presence of *bar* gene located on the antisense construct. Out of 45 putative transgenics tested, 30 were found to contain the transgene, as confirmed by the amplification of 294 bp fragment of the *bar* gene (Figure 3). The T_1_ generation progenies (15–20 plants) of the confirmed 27 plants were raised in glasshouse. On PCR analysis, some of them did not show the presence of the *bar* gene indicating segregation of the transgene.

**Figure 4:**
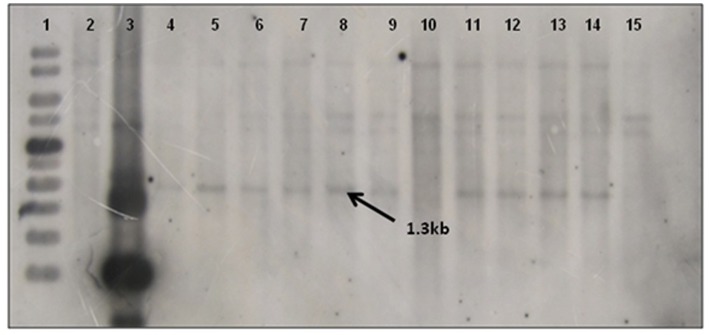
Southern blot of T_0_ Transgenics lines for gene integration using pJS108-*CYP79A1-AS* gene as probe. The genomic DNA of T_0_ plants was digested with *Hind*III enzyme and blot was probed with radiolabelled pJS108-*CYP79A1-AS* gene. **Lane 1**- GeneRuler 1Kb (Fermentas), **Lane 2**- Wild type genomic DNA (negative control), **Lane 3**- Plasmid DNA of pJS108-CYP79A1-AS construct used for transformation, **Lanes 4–15** indicate the line number of respective T_0_ plants (S28, S13, S6, S35, S4, S32, S15, S22, S25, S2, S9, S7). The presence of 1.3 Kb band indicates the presence of transgene (pJS108-CYP79A1-AS gene).

**Figure 5:**
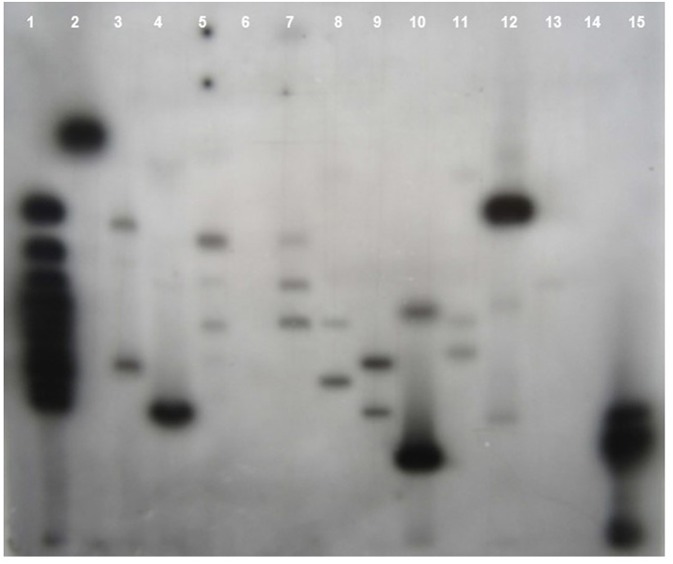
Southern blot of T_0_ Transgenics lines for sites of transgene integration. The genomic DNA of T_0_ plants was digested with *Xba1* enzyme and blot was probed with radiolabelled *bar* gene. **Lane 1**- GeneRuler 1Kb DNA ladder (Fermentas), **Lanes 2–13** indicate the line number of respective T_0_ plants (S13, S18, S4, S32, S17, S6, S35, S15, S25, S8, S16, S34), **Lane 14**- Wild type genomic DNA (Negative control), **Lane 15** Plasmid DNA of pJS108-CYP79A1-AS construct used for transformation as positive control.

Southern blot hybridization was carried out using 20 μg of genomic DNA. To confirm the presence of transgene, the *Hind*III enzyme that releases the major fragment (1.3 kb) of the *CYP79A1* antisense gene was deployed. The integration of the transgene could be confirmed in 27 of the 30 transgenics as evident from the 1.3 kb fragment lighting up in the *Hind*III restricted genomic DNA of the transgenics compared to the DNA from non-transformed control plant (Figure 4). Southern analysis was also used to probe the number of sites of integration of the transgene in the transgenic plant DNA. This was done in plants that were confirmed for the presence of transgene through *Hind*III restriction analysis. A single restriction enzyme *Xba*I, that cuts the plasmid only once at a specific site in the transgene, was employed (Figure 5). Once the transgene is integrated into the genome at a particular site, the restriction analysis performed using such enzyme would lead to generation of fragments of varying length since the other end of the fragment would be at a random site in the plant genome. It was observed that the plants exhibited 2–5 sites of integration as visualized by the fragments that showed up by hybridizing with the transgene *bar* probe. Though large proportion of plants exhibited 2 to 3 sites of transgene integration, some of them had up to five sites of integration. The Plants S13 and S34 showed one fragment followed by S15 and S35 with two fragments. While S6, S8, S18, and S25 exhibited three fragments, at least four fragments were seen in the plant S4, and five fragments could be observed in S16 and S32. Each fragment indicated one site of transgene integration and thus multiple sites of integration were observed in these transgenics. Each site of integration may carry one or more copy of the transgene.

#### Reverse Transcription (RT-PCR) Based Confirmation

**Figure 6:**
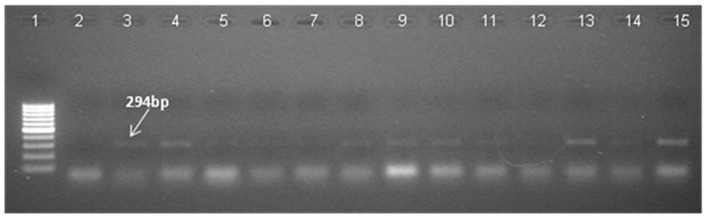
Reverse transcriptase PCR (RT-PCR) of T_0_ Transgenics lines for *bar* gene RT-PCR with primers specific to *bar* gene shows amplification of 294 bp product. Lane **1** signifies 100 bp DNA ladder (Fermentas). **Lane 2** designate negative control. Wild type RNA of untransformed plant was used as negative control, **Lanes 3–15** indicate the line number of respective T_0_ plants (S18, S28, S13, S6, S35, S4, S32, S17, S22, S11, S25, S9, S16). The presence of 294 bp band indicates the expression of *bar* gene in transgenics plants.

**Figure 7:**
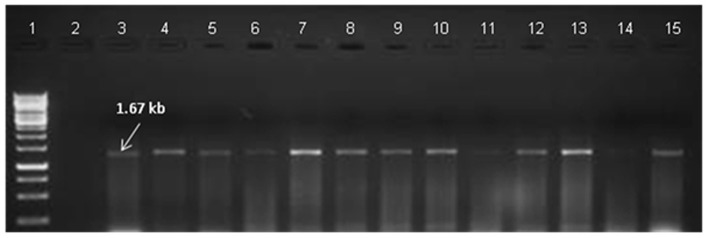
Reverse transcriptase PCR (RT-PCR) of T_0_ Transgenics lines for antisense gene. RT-PCR analysis of To Transgenics lines using antisense *CYP79A1* gene-specific primers shows amplification of 1.67 Kb product. **Lane 1** signifies GeneRuler 1 kb DNA ladder (Fermentas), **Lane 2**- designate negative control. Wild type RNA of untransformed plant was used as negative control; **Lanes 3–15** indicate the line number of respective T_0_ plants. (S18, S28, S13, S6, S35, S4, S32, S17, S22, S11, S25, S9, S16).

RT-PCR of transgenics was done in 30 PCR confirmed T_0_ plants. RT-PCR using the *bar* primers revealed that 27 transgenics expressed the transgene in the events tested (Figure 6). *Bar* gene-specific RT-PCR product sequence matched the known sequence of the marker gene. RT-PCR was also done to amplify the *CYP79A1* antisense cDNA product. The reverse sequence of the native gene portion was recovered by sequencing the product of antisense *CYP79A1***-**specific RT-PCR (Figure 7).

#### Visualized Selection Marker Phenotype Through Leaf Painting Assay

Identification of the functionality of the transgene in the transgenic plants and tracking the inheritance of the transgenes in their progeny was done through leaf painting assay. Seeds from 36 T_0_ transgenic plants along with non-transformed controls were germinated. Leaf painting assay were performed in 25 days old plants with 0.05% PPT concentration. Progenies from 36 T_0_ plants were screened by PPT leaf painting of which 20 progenies (S10, S11, S16, S22, S23, S29, S32, S33, S36, S5, S8, S12, S18, S20, S4, S24, S25, S27, and S26) were without chlorosis in at least 50% plants.

### Developed Transgenic Events Showed Reduced HCN Level in the Advanced Generations (T_0_, T_1_, T_2_, T_3_)

The HCN content was determined in T_0_ transgenics 45 days after transferring them to pots. The HCN content was reduced in the confirmed T_0_ transgenic plants which ranged from 17.25 to 178.66 μg/g with a mean of 104.06 μg/g as compared to 192.08 μg/g in the non-transformed control. There were 14 transgenic plants with <100 μg/g HCN and another 13 possessed more than 100 μg/g (Table 1). Glasshouse-raised T_1_ progenies of 24 events were assayed for HCN content in leaves 45 days after sowing. Each event was assayed along with non-transformed control. Events with a greater proportion of plants exhibiting substantially reduced HCN content were observed. The HCN content in T_1_ progenies varied from 5.1 to 149.8 μg/g as against mean HCN content of non-transformed control of 211.5 μg/g. Antisense mediated down-regulation of *OMT* gene in maize resulted in significantly lower *O*-methyltransferase activity in T_1_ transgenics with some plants showing a 60% reduction (16).

**Table 1:** Frequency distribution of *CYP79A1* antisense transgenics (T_0_) for HCN content.

**Class range (HCN in μg/g)**	**0–20**	**21–40**	**41–60**	**61–80**	**81–100**	**101–120**	**121–140**	**141–160**	**161–180**	**181–200**
No. of transgenics	1	1	4	3	5	1	5	3	4	0

*HCN **(**μg/g) in (i) non-transformed control = 192.08 ± 2.15 (n = 20); (ii) Transformed (but no gene integration was detected) control = 207.0 (n = 20)*.

T_1_ generation plants confirmed by PCR were characterized for HCN production and those with lowered HCN were advanced to next generation. The progenies (T_2_) of the promising 43 T_1_ plants were characterized by PCR and assayed for HCN as earlier for advancement to T_3_. The plants with reduced HCN consistently across the generations were identified (Table 2). T_1_ progeny of S4 had minimum HCN of 50.3 μg/g and maximum of 156 μg/g with a mean HCN of 88.3 μg/g. Its progeny S4-2 showed 88.2 μg/g of HCN which was further advanced to T_2_. Its progenies exhibited HCN levels from 11.5 to 131.0 μg/g. Similarly, in the S35 event HCN content was 132.6 μg/g while in T_1_ progeny it ranged between 64.3 and 326.2 μg/g. From this event, 2 progenies (S35–10 and S35–12) were advanced to T_2_ which had HCN ranges of 2.8- 133.7 and 16.0- 154.8 μg/g. T_3_ generation is an advanced stage where lines are expected to be relatively stable for inheritance and transgene expression. Hence, 7T_2_ plant progenies from two T_0_ plants (S4 and S32) that were consistently positive in molecular analyses and possessed lowered HCN content across generations were advanced to T_3_. The progenies of individual T_0_ plants viz., S4, S6, S10, S17, S3, and S36 showed Mendelian segregation (3:1 ratio) for HCN content (lower than control: higher than control). For the hemizygous transgene, T_1_ progeny segregated in 3:1 ratio for HCN content.

**Table 2:** HCN content (in μg/g) in promising events in T_1_ and T_2_ generations.

**S No**.	**Event (T_**o**_)**	**HCN content in T_**o**_**	**HCN content in T**_****1****_ **progeny**			**Selected T_**1**_ plant ID**	**HCN in selected T_**1**_ plant**	**HCN content in T**_****2****_ **progeny**		
			**Minimum**	**Maximum**	**Mean**			**Minimum**	**Maximum**	**Mean**
1	S4	154.7 ± 7.2	50.3	156.0	88.3	S4-2	88.2	11.5	131.0	55.5 ± 7.2
2	S16	68.12 ± 17.5	5.1	112.4	57.4	S16-2	70.9	36.6	171.5	113.9 ± 28.5
3	S17	60.8 ± 19.8	33.5	169.1	104.9	S17-6	68.6	4.0	191.9	92.2 ± 26.7
4	S20	115.0 ± 23.3	53.1	174.4	97.4	S20-3	71.3	0.5	160.1	50.5 ± 24.1
5	S25	46.8 ± 37.4	30.7	295.1	144.5	S25-15	50.3	4.7	99.9	42.4 ± 23.5
6	S26	87.4 ± 38.8	68.7	370.0	151.6	S26-7	68.7	9.5	106.9	38.4 ± 26.4
7	S32	86.6 ± 39.6	51.2	209.3	104.2	S32-1	51.2	8.6	134.8	66.5± 26.4
8	S33	139.1 ± 45.7	56.7	495.2	292.5	S33-4	100.0	2.3	150.3	55.7 ± 22.3
9	S35	132.6 ± 28.3	64.3	326.2	198.2	S35-10	94.7	2.8	133.7	43.3± 28.0
10	S35	–	–	–	–	S35-12	67.9	16.0	154.8	88.3 ± 27.0
11	Control^*^	192.08	89.3	399.0	213.1	Control	211.5	100.1	295.0	189.1 ± 18.5

* *Each of the T_1_ and T_2_ progenies were compared against separate non-transformed controls and the range and mean across all those controls is given above*.

### Phenotyping of T_3_ Generation of Transgenics Plants Having Reduced Level of Hydrogen Cyanide

A total of seven T_2_ plant progenies from S4 and S32 transgenic events that consistently possessed lower HCN content across generations were evaluated in T_3_ generation. More than 90 percent of the T3 progenies were positive for presence of transgene as indicated by PCR (data not shown) suggesting that advancing of generations had effectively eliminated the non-transgenic sergeants.

The HCN analysis done in 45 days old plants showed consistently low HCN levels in these plant progenies (Table 3). The level of HCN ranged from 6.1 μg/g (in S4-26) to 107.8 μg/g (in S4-25) with a mean of 62.9 μg/g compared to a range of 184.2–398.1 μg/g in control with a mean of 221.4 μg/g. In the progenies of S32 segregants, the range was 25.1 μg/g (in S32–21) to 150.2 μg/g (in S32–21) with a mean of 76.2 μg/g. Thus, the reduced cyanogen potential of T_1_ plants was inherited stably in these progenies. It is proposed that a total of 3–5 progenies with lowest HCN content from each event may be advanced to next generation as they are expected to be the most promising for lower cyanogenic potential.

**Table 3:** HCN content in T_3_ progenies of transgenics (in μg/g).

**S. no**.	**Plant ID**	**Range (reduced HCN)**	**Mean**	**Control (mean)**	**No. of plants**
1	S32-12	32.4–96.9	63.2 ± 9.1	217.2 ± 3.6	18
2	S32-14	13.7–127.8	82.3 ± 5.7	200.7 ± 2.4	19
3	S32-21	25.1–150.2	75.4 ± 2.4	196.9 ± 13.4	8
4	S4-25	13.7–107.8	53.8 ± 9.3	240.8 ± 3.6	11
5	S4-26	6.1–104.3	60.9 ± 6.0	187.0 ± 19.7	16
6	S4-21	49.3–82.9	67.3 ± 17.0	216.8 ± 17.6	13

### Reduction of *CYP79A1* Gene Expression in Developed and Stable Transgenic Plant Progenies

**Figure 8:**
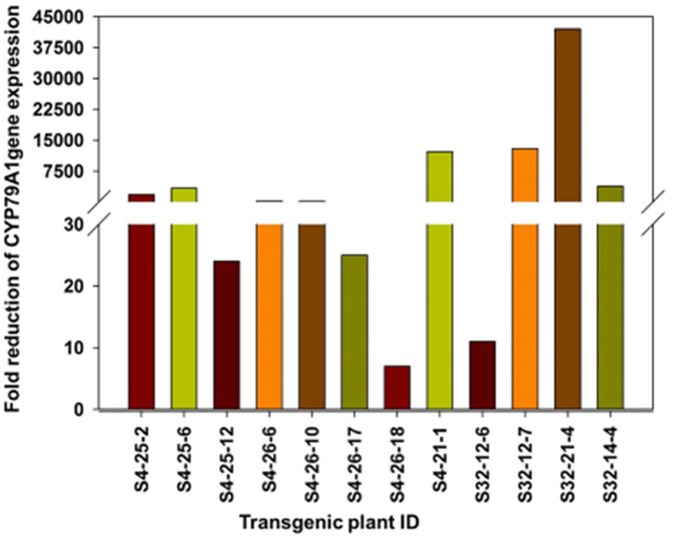
Fold reduction (on log scale) of *CYP79A1* gene expression as determined by quantitative Real time PCR in selected progenies of two promising events S4 and S32. The plants from the event S4 and S32 showed reduced expression of the CYP79A1.

The reduction of *CYP79A1* gene expression in the transgenic plant progenies was assayed through quantitative PCR (Real-time PCR) in selected progenies of two promising events S4 and S32 (Table 4; Figure 8). The plants from the event S4 showed from 7 to 12,180 times reduced expression of the *CYP79A1* (Table 4). Similarly, the reduction was from 11 times to 42,017 times in the plants of event S32. Both events had recorded lower HCN levels in the range of 6–84 μg/g. The correlation of the HCN content with normalized Cp ratio was statistically significant (Pearson product-moment correlation *r* = 0.532, *p* < 0.0375). Therefore, it can be concluded that the reduced HCN levels in the transgenics are due to the lowered expression of *CYP79A1* gene.

**Table 4:** Reduction in *CYP79A1* gene levels as estimated by quantitative PCR.

**Sample no**.	**Progeny ID**	Target/reference Cp ratio^*^(normalized)	**Reduction in *CYP79A1* levels (number of folds)**	**HCN (μg/g)**
1	S4-25-2	0.00058	1,737	17
2	S4-25-6	0.00030	3,342	14
3	S4-25-12	0.04092	24	25
4	S4-26-6	0.00494	203	23
5	S4-26-10	0.01033	97	6
6	S4-26-17	0.03951	25	56
7	S4-26-18	0.15234	7	84
8	S4-21-1	0.00008	12,180	57
9	S32-12-6	0.09238	11	32
10	S32-12-7	0.00008	12,903	51
11	S32-21-4	0.00002	42,017	25
12	S32-14-4	0.00026	3,824	27

* *Control (non-transformed) ratio was considered as unity and relative expressions were worked out*.

## Discussion

Sorghum (*Sorghum bicolor* L. Moench) is grown extensively throughout the world owing to its wider adaptability for varied soil and moisture conditions and utility as food, fodder, fuel, and fiber. Mixed crop and livestock farming systems are mostly prevalent in India. Therefore, both grain and fodder are of equal importance, underscoring the interdependence of crops and livestock. Sorghum is an ideal forage crop due to its quick growth, high yielding ability (350–400 q/ha and 700–750 g/ha of green fodder from single cut and multi-cut varieties/hybrids, respectively), high dry matter content (25–30%), better quality (crude protein 5–9%) and its suitability for various forms of utilization like green chop, silage and hay.

The particle inflow gun was deployed in the present investigation for sorghum transformation using the plasmid carrying the *CYP79A1* antisense strand under the control of *Actin1* promoter (Figure 1). The protocols for producing transgenic sorghum are still being standardized using various methods of DNA transfer (25, 26). Genetic transformation of sorghum using shoot meristem explants was carried in this experiment (27, 28). The advantage of adopting shoot meristem explants (Figure 2A) for the transformation lies in its year-round availability unlike explants such as immature embryos and immature inflorescences (27, 29–31). This system could be conveniently adapted for sorghum transformation as large number of multiple buds and somatic embryos capable of regeneration are obtained in a relatively short period. Heterogeneity of the explant is also minimized as the shoot apices have very limited mother plant tissue, which also resulted in enhancing the rate of multiplication.

Thirty putative transgenics were confirmed by showing the amplification of 294 bp fragment of the *bar* gene in the generated transgenics. Transformed tissues with the selective marker gene may be capable of detoxifying the selective agent so efficiently that non-transformed tissue in close proximity can also survive (32) and such a phenomenon can lead to false positives during *in vitro* selection. Out of 30 PCR confirmed transgenics, 27 showed the transgene integration. In the transgene integration southern blot, upper fragments that appear in all plants including control indicated the native *CYP79A1* DNA. Similar results were obtained in cassava (12). The Plants S13 and S34 showed single copy insertation and comparatively low dhurrin expression, but due to the unavailability of seeds, we could not advance these lines. Interestingly, as the data of HCN content in T_1_ reveal, plants with more sites of integration such as S4, S16, and S32 (4–5 sites of integration) had lower HCN content. However, in progenies of plant S35 (2 sites of integration), some plants did exhibit higher HCN content. The integration of multiple copies of transgene have been reported by several workers using the biolistic transformation method (33) observed that in rice, the higher copy number of transgenes led to its higher expression levels, where more β-carotene was produced. Similarly, transgenic rice with more than one copy of *Bt* gene performed better in field conditions suggesting effective levels of transgene expression (34, 35). Also, observations of high transgene expression levels were reported in wheat transformed with multiple copies of reporter genes (36). Transformants selected for producing higher levels of pharmaceuticals were found to contain three or more transgene copies (37). Besides, events with a single copy losing the expression of transgene in T_1_ and later generations have also been observed (38). Transgene “switching off” is largely due to the presence of one or more rearranged copies rather than multiple copies of the transgene (39). However, on the contrary, multiple copies of transgenes were thought to result in gene silencing due to co-suppression (40). It was also believed that the expression of a multi-copy transgene may vary over generations compared to a low copy number transgene event (41). The important problem for the potential use of transgenic plants for crop improvement is the instability of transgene expression. The expression of the integrated transgene was confirmed by reverse transcription PCR (RT-PCR). Few of the T_0_ regenerants in the present study failed to show up in RT-PCR, but were found positive in PCR and/or Southern for transgene(s). They exhibited HCN levels similar to that of control (186–219 μg/g). Gene silencing and interactions between multiple copies of the same transgene or different transgenes are known to result in unexpected expression patterns of foreign genes (42). This transgene inactivation in T_0_ plants maybe because of the hemizygous state of the transgene (43). Methylation can be induced by interactions between homologous transgene copies or may reflect genomic position effects (32). The gene silencing may occur at transcriptional (44), post-transcriptional levels (45), and reported a functional role for methylation in gene silencing (42).

The leaf painting assay also confirmed the presence of the *bar gene* by showing various ranges of chlorosis in 36 T_0_ progenies. Out of 36 putative transgenics, 20 progenies were without chlorosis in plants. The progenies of T_0_ plants that showed low HCN content also showed resistance to PPT indicating the expression of both *bar* and *CYP79A1* antisense transgenes in these plants. The assay was also successfully used to confirmed transgenic integration in Indicia rice (46).

Antisense approach had been successful in down-regulation of several pathways in plant species. Transgenic tomatoes were developed with reduced sucrose synthase (SuSy) activity in fruit by expressing an antisense RNA fragment of the *TOMSSF* gene under the control of the cauliflower mosaic virus 35S promoter (47). The transgenic citrus lines that produce higher level (over expression) of antisense ACS RNA were found to repress the increase of ACC content following the chilling treatment (48). Cassava transgenic were also developed with reduced levels of *CYP79D1* and *CYP79D2* enzymes resulting in the inhibition of cyanogen production by antisense technology (12). Other robust tools such as RNA interference-mediated down-regulation technologies (14) were not deployed since it was not intended to completely block the dhurrin biosynthesis pathway. The synthesis of a small quantity of the dhurrin may be desirable as a defense against insects (49, 50). Antisense technology is known to substantially down-regulate but not to block the target gene completely (12, 16, 17). Generated transgenics were also showed the Mendelian segregation for HCN content (lower than control: higher than control). Earlier researchers have indicated that hemizygous nature of transgenics and the transgene segregation in 3:1 ratio of dominant: recessive in selfed progeny are common (51, 52). Transgene expression heterogeneity may also be due to the influence of factors like position effects, gene rearrangement, gene silencing and co-suppression (43, 51, 53).

## Conclusions

In the present study, the antisense RNA approach was used to down-regulate dhurrin synthesis. The inheritance of the reduced HCN content was studied up to T_3_ generation by advancing the progenies based on screening for transgene by PCR and assaying the HCN content. The HCN content in the transgenics varied from 5.1 to 149.8 μg/g compared to 192.08 μg/g in the non-transformed control on dry weight basis. Progenies with reduced HCN content were advanced after each generation till T3. Other robust tools such as RNA interference-mediated down-regulation technologies were not deployed since it was not intended to completely block the dhurrin biosynthesis pathway. The synthesis of a small quantity of the dhurrin may be desirable as a defense against insects. This investigation demonstrated that the antisense *CYP79A1* strategy is effective in producing sorghum plants with lower cyanogenic potential. The frequency and inheritance of the transgene improved with the advancement of generations perhaps due to elimination of non-carrier plants of transgene by molecular and HCN analyses. The rice *actin1* promoter is adequately driving the antisense *CYP79A1* expression in the leaf tissues of sorghum. Absence of other unintended agronomic variations in the transgenics (in terms of fodder and grain yield components; data not shown) indicated that the transgenics thus obtained would be useful as cultivars. The present study effectively demonstrated that the antisense strategy was effective in producing sorghum plants with low cyanogenic potential and the biolistic method of transformation remains a useful tool for obtaining transgenic sorghum plants.

## Data Availability

The raw data supporting the conclusions of this manuscript will be made available by the authors, without undue reservation, to any qualified researcher.

## Author Contributions

AP and BB conceived and designed the experiments, analyzed the data, and wrote the paper. AP and PM performed the experiments.

### Conflict of Interest Statement

The authors declare that the research was conducted in the absence of any commercial or financial relationships that could be construed as a potential conflict of interest.
