# Supplementary figures and images for: Down-Regulation of CYP79A1 Gene Through Antisense Approach Reduced the Cyanogenic Glycoside Dhurrin in [Sorghum bicolor (L.) Moench] to Improve Fodder Quality
Source: Front Nutr. 2019 Aug 30;6:122. doi: 10.3389/fnut.2019.00122 (PMC6729101; doi:10.3389/fnut.2019.00122)

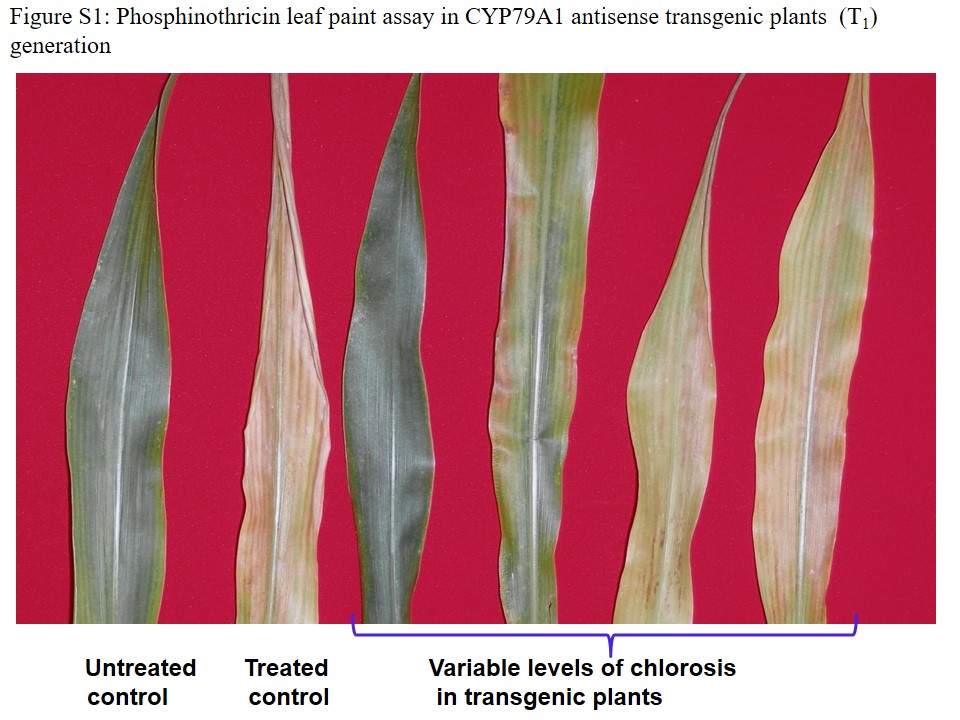

Supplement: Supplementary file 1 [file Image_1.JPEG]
